# Design and implementation of high sampling rate and multichannel wireless recorder for EEG monitoring and SSVEP response detection

**DOI:** 10.3389/fnins.2023.1193950

**Published:** 2023-06-29

**Authors:** Ruikai Li, Yixing Zhang, Guangwei Fan, Ziteng Li, Jun Li, Shiyong Fan, Cunguang Lou, Xiuling Liu

**Affiliations:** ^1^The College of Electronic Information Engineering and the Hebei Key Laboratory of Digital Medical Engineering, Hebei University, Baoding, China; ^2^Information Center, The Affiliated Hospital of Hebei University, Baoding, China

**Keywords:** EEG, SSVEP, BCI, RHS2116, inflatable helmet, electroencephalography

## Abstract

**Introduction:**

The collection and process of human brain activity signals play an essential role in developing brain-computer interface (BCI) systems. A portable electroencephalogram (EEG) device has become an important tool for monitoring brain activity and diagnosing mental diseases. However, the miniaturization, portability, and scalability of EEG recorder are the current bottleneck in the research and application of BCI.

**Methods:**

For scalp EEG and other applications, the current study designs a 32-channel EEG recorder with a sampling rate up to 30 kHz and 16-bit accuracy, which can meet both the demands of scalp and intracranial EEG signal recording. A fully integrated electrophysiology microchip RHS2116 controlled by FPGA is employed to build the EEG recorder, and the design meets the requirements of high sampling rate, high transmission rate and channel extensive.

**Results:**

The experimental results show that the developed EEG recorder provides a maximum 30 kHz sampling rate and 58 Mbps wireless transmission rate. The electrophysiological experiments were performed on scalp and intracranial EEG collection. An inflatable helmet with adjustable contact impedance was designed, and the pressurization can improve the SNR by approximately 4 times, the average accuracy of steady-state visual evoked potential (SSVEP) was 93.12%. Animal experiments were also performed on rats, and spike activity was captured successfully.

**Conclusion:**

The designed multichannel wireless EEG collection system is simple and comfort, the helmet-EEG recorder can capture the bioelectric signals without noticeable interference, and it has high measurement performance and great potential for practical application in BCI systems.

## 1. Introduction

In recent years, with the continuous innovation in computer technology, electronic information, and neuroscience, brain–computer interface (BCI) technology has witnessed rapid development (Zhou et al., 2020; Gao et al., 2021). The electroencephalogram (EEG)-based BCI system refers to the acquisition of weak original EEG signals from the brain and displays them through an EEG shape map to facilitate the analysis, processing, and interactive control of subsequent signals. As a non-invasive monitoring method, multi-channel EEG is widely used for diagnosing and researching nervous diseases, such as migraine, epilepsy, and brain tumors (Do Valle et al., 2016; Daoud and Bayoumi, 2019). In recent years, with the advance of technology, EEG devices tend to be portable and compact, forming various wearable EEG systems (He et al., 2015; Jiang et al., 2017; Holtze et al., 2022). Meanwhile, the demand for wearable EEG systems for BCI and out-of-the-lab applications is rising. In a portable EEG system, the signal acquisition, processing, and transmission circuit are integrated and installed on some accessories, such as a hat or a headset (Chen et al., 2015; Zhao et al., 2018). The vital portability and high comfort make it more suitable for dynamic applications (Haueisen et al., 2017; Schirrmeister et al., 2017; Dilena et al., 2021; Zanetti et al., 2021).

Most of the existing EEG recording systems use wired connections, Bluetooth-based or Wi-Fi-based wireless interfaces with a sampling rate of up to l kHz or less. Such configuration is sufficient for most basic EEG applications, but it cannot meet the requirements of some clinical practice scenarios, such as auditory brainstem response (ABR) and neural spikes recording; the frequency band of these signals is typically in the range of a few kilohertz; thus, most of the existing wireless wearable EEG recorders are not capable of capturing these signals. In addition, scalp EEG has been widely used in many areas due to its convenience and effectiveness, as the “gold standard” of EEG acquisition. The wet electrode has the advantages of low impedance, high stability, and high signal-to-noise ratio (SNR), and it can provide a reliable EEG signal (Wang et al., 2021). However, the installation process of wet electrodes is complex, and the experimental preparation process is time-consuming. Hence, the wet electrode is not very suitable for long-term and ambulatory applications. Dry electrodes allow more freedom in the design and fabrication of portable EEG systems and can be self-applied without preparation. Recently, some researchers have proposed using self-made dry electrodes to collect EEG signals (Nathan and Jafari, 2015; Liu B. et al., 2021) as this can simplify the preparatory work and significantly improve the portability of the EEG acquisition system.

However, the contact impedance of dry electrodes is generally greater than that of the wet electrode, and there are still great challenges in the portable installation and wearing of dry electrodes, and the adjustment of contact impedance between electrodes and the scalp. Many research groups have been committed to this research. Lee et al. (2018) proposed a dry electrode-based portable 16-channel EEG recorder, employing spring-loaded probes to maintain constant pressure on the surface of the uneven scalp. This method is convenient and easy to operate, but the contact pressure depends on the stiffness coefficient of the spring, and it cannot be adjusted arbitrarily. Fiedler et al. developed a polymer-based multipin dry electrode with an embedded force sensor, applying regular forces of 2N or more to reduce the electrode-skin impedance (Fiedler et al., 2018). However, adding a pressure sensor on each electrode leads to increased cost and volume. Yu et al. (2016) developed a portable 32-lead EEG acquisition system based on the inflatable helmet and dry electrode. Using spring-loaded dry electrodes and an inflatable airbag makes the wearing process simpler, more comfortable, and more convenient than traditional EEG systems. The inflatable airbag can be used to increase the contact force between the electrode and the scalp, and the contact impedance can be reduced by increasing the air pressure of the airbag. However, in this design, the inflatable airbag was divided into several local areas, so the contact force of each electrode was not able to be very even, the range of electrode activity was small, and the position was easily affected by surrounding electrodes. Moreover, a Bluetooth-based wireless interface was adapted to transmit EEG data sampled at 500 Hz, and such configuration is sufficient for most basic EEG applications but cannot meet the special requirements for high accuracy sampling rate, such as measurement of the auditory-brainstem response and neural spikes signals.

In this study, to address the problems mentioned above, a wearable wireless inflatable helmet-EEG recorder was designed and implemented. The developed EEG recorder is mainly composed of three major sub-systems: (1) an inflatable helmet with dry electrode for contact impedance adjustment; (2) a wearable wireless multi-channel EEG signal collecting device with a high sampling rate and high transmission rate; and (3) data reception, preprocessing, and display realized by upper PC software. The helmet was designed as a hollow structure with 32 independent inflating airbags to ensure each electrode could connect well with the scalp. A highly integrated chip RHS2116 dedicated to handling EEG signals was combined with ARM and FPGA for high-precision and high-speed data acquisition. The experimental results demonstrate that the proposed helmet-EEG recorder performed well in signal acquisition and is feasible for steady-state visual evoked potential (SSVEP)-based BCI applications.

## 2. EEG helmet

### 2.1. Structural design of EEG helmet

**Figure 1:**
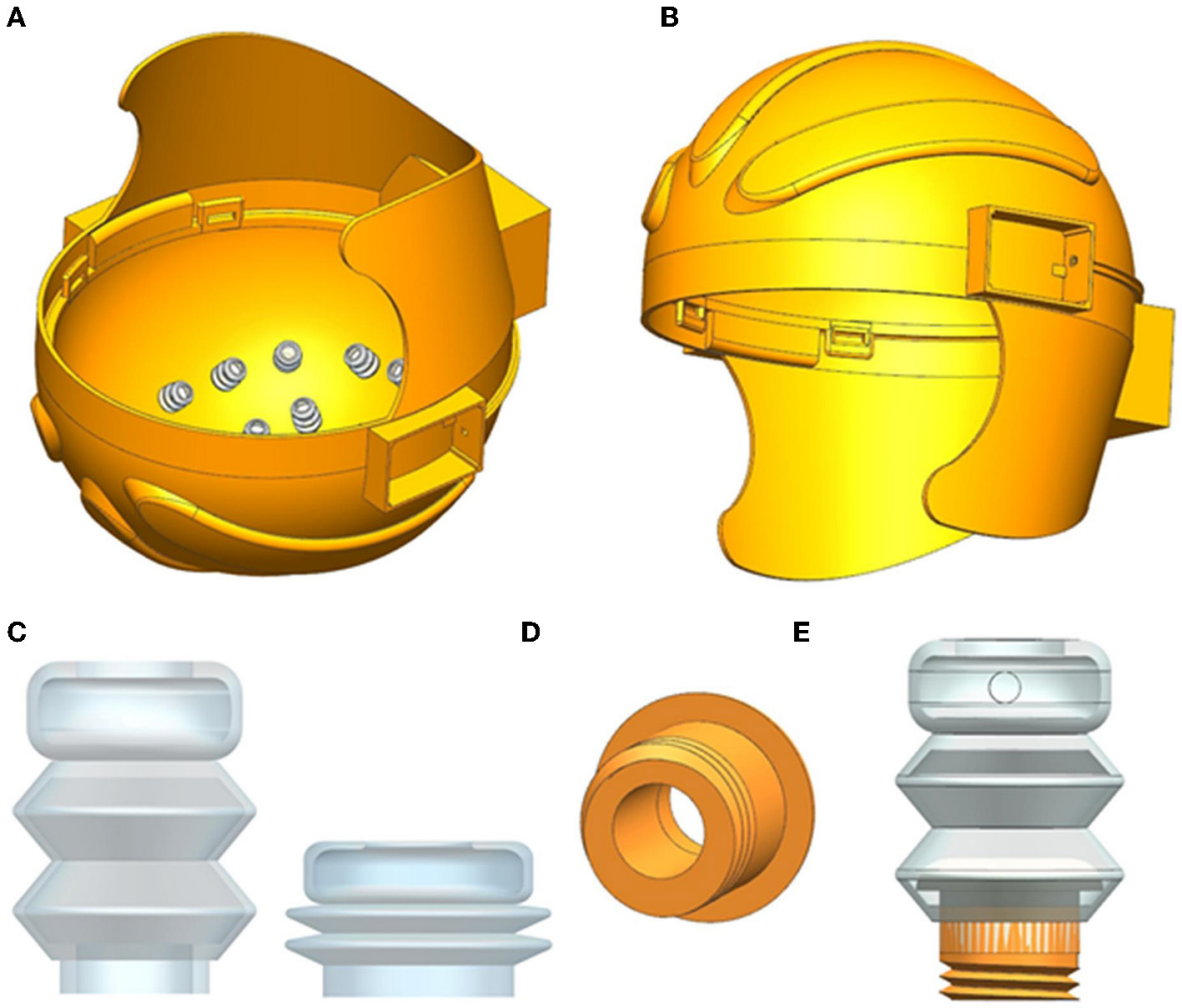
**(A)** Inflatable helmet structure diagram, **(B)** the side view of inflatable helmet diagram, **(C)** inflatable airbag before and after inflation, **(D)** structure diagram of the connector, and **(E)** diagram of airbag combined with connector.

The inflatable helmet proposed in this study includes 3D-printed hollow helmets, inflatable airbags, and dry electrodes, which are accessible to the user without the complex operation of a traditional EEG cap, as shown in Figures 1A, B. The helmet shell of the inflatable helmet is modeled using UG software, and a sandwich structure is designed to provide the air circuit. The helmet's inner shell opens the M10 threaded hole, which is distributed according to the international standard EEG electrode distribution mode. In Figure 1C, the airbag material used in this study is silica gel, a highly stable material with a specific mechanical strength. Due to silica gel's good elasticity and biocompatibility, the subject feels more comfortable than with the traditional EEG cap, which can make the collected EEG signal more accurate.

Due to the difficulty in the connection between the flexible inflatable airbag and the helmet, we considered using 3D printing hollow components to realize the connection between them, as shown in Figure 1D. One end of the component has an external M10 thread, the other end has a convex surface to connect the inner wall of the airbag, and its hollow structure ensures air circulation between the helmet and retractable airbag, as shown in Figure 1E. The dry electrode was fixed to the top of the airbag. When inflating air into the helmet, the expansion of the airbag will change the contact force between the electrode and the scalp.

The circuit board for multi-channel EEG recording is arranged in the rear end of the helmet, and the left and right ends are placed with an air pump and an inflation control unit, which increases the aesthetics of the inflatable helmet. The inflatable airbag is designed with up-and-down telescopic and flexible disassembly to facilitate the distribution of electrodes as needed, allowing the increase or decrease of signal channels as desired. The designed inflatable helmet-EEG recorder can support 8/16/32 channel acquisition and can be further expanded to more channels.

### 2.2. Implementation of inflatable helmet

**Figure 2:**
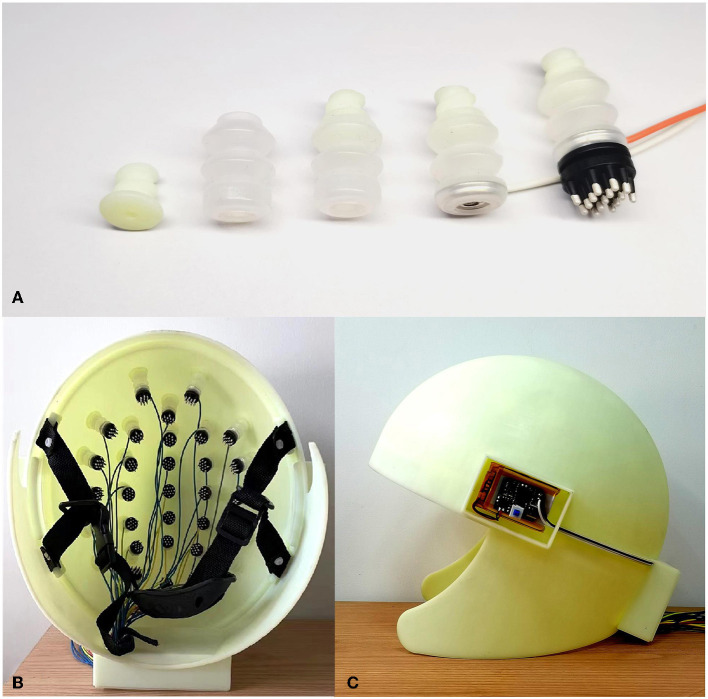
**(A)** Components and assembly process of the inflatable airbag, **(B)** photography of the inside of the assembled inflatable helmet, and **(C)** side view of the helmet with inflation control circuit board.

The model of the inflatable helmet and connector was manufactured using 3D printing technology, and the airbag was prepared by compression molding. As illustrated in Figure 2A, the inflatable airbag material is soft silicone gel, and the hollow helmet and connector are printed using tough resin. The airbag is designed as a spring shape to ensure sufficient scalability. At the end of the airbag, a dry electrode was fixed with a buckle structure. The dry electrode employed in the study is DX-S02A (Greentek, China), and it provides 15 prongs and a standard male snap button, each prong having a length of 5 mm. The prong design can overcome the obstruction by the hair and provide adequate scalp contact that does not hurt the skin. The impedances of the electrodes were maintained at levels less than 500 Ω. The combination of 32 inflatable components and the helmet is shown in Figures 2B, C.

**Figure 3:**
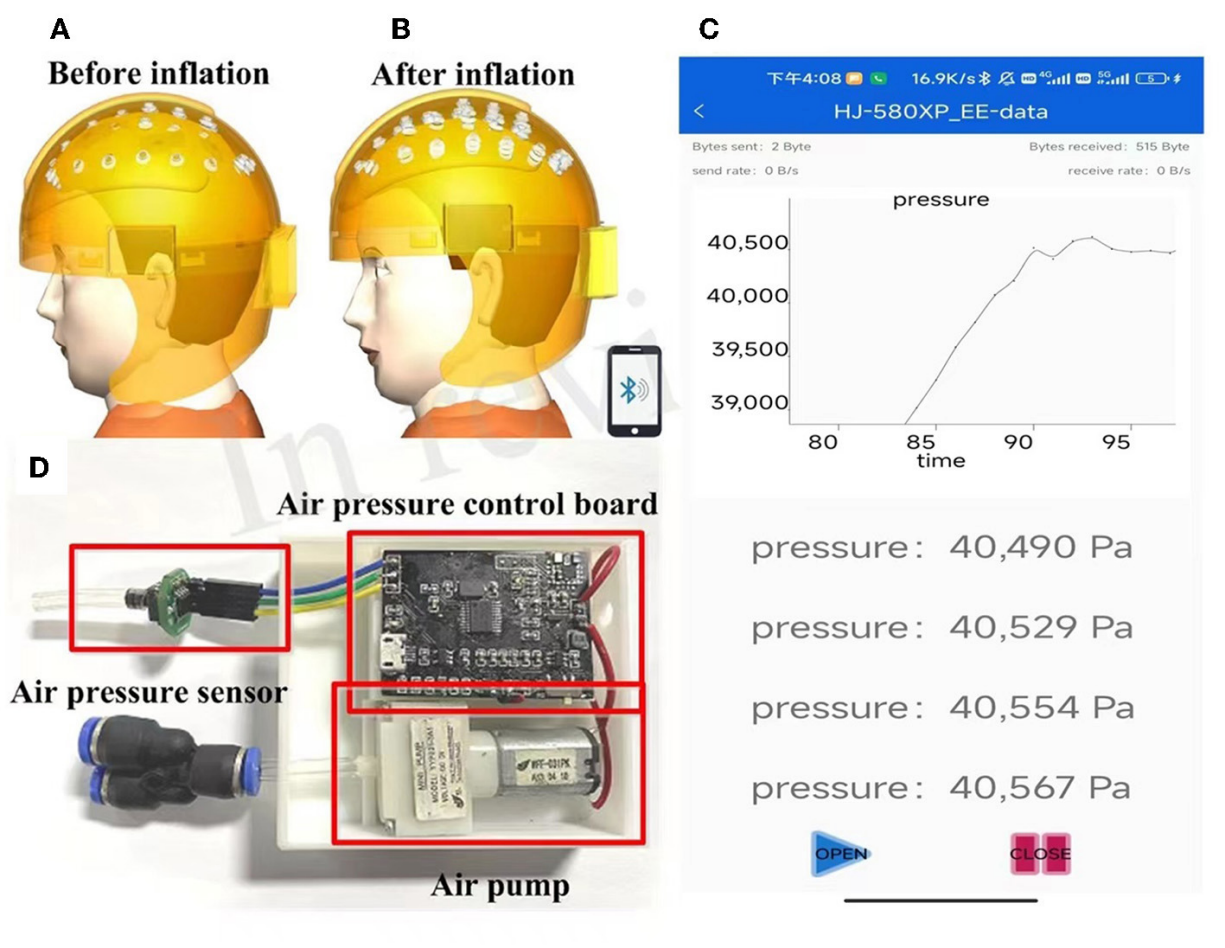
Renderings of inflatable EEG helmet before **(A)** and after **(B)** inflation, **(C)** circuit board for inflating control, and **(D)** real-time monitor of the air bag pressure on a mobile APP.

Figures 3A, B shows 3D renderings of the helmet before and after airbag inflation. When the airbag is inflated, it can press each electrode more firmly into the scalp, improving the contact between the electrode and the scalp. As illustrated in Figure 3C, a silicon pressure sensor (XGZP6847A, CFsensor Company) is employed to detect the air pressure of an inflatable helmet, the detection accuracy is 0.5 kPa, and the operating range is 0–100 kPa. The micro air pump employed in the system is YYP031-3A1, its volume is 42.2 mm^*^14 mm^*^12 mm, and the operation voltage is 3.3 V. Experiment results show that the air pump can inflate the helmet to a maximum air pressure of 55 kPa. A mobile APP was developed to monitor the air pressure inside the helmet, as shown in Figure 3D. Bluetooth communication is adopted between the mobile communication terminal and the inflation control circuit board. The timer interrupt is used to control the acquisition of ADC and the switch of the air pump, the air pressure value is displayed on the mobile APP in real time, and the wearer can adjust the pressure according to personal and experimental needs. In addition, to ensure the safety of the wearer, the shutdown threshold was set to 50 kPa to stop inflation.

It has been verified that the airbag can expand by approximately 1.5 cm when the inflation pressure reaches 50 kPa. The inflating airbag presses the dry electrode to the scalp, and the airflow within the sandwich-structured helmet ensures that each electrode is evenly pressurized, and insufficient pressure or excessive pressure on a single electrode is unlikely to happen. The airbag and air circuit are well-sealed and can maintain no air leakage, and a separate solenoid valve is used for deflation and decompression. The inflatable helmet-EEG recorder proposed in this study has the advantage of convenient preparation before the experiment, adjustable electrode-scalp contact pressure, and high comfort for the subject in the test process.

## 3. High-performance data acquisition

### 3.1. Design of high-speed wearable EEG system

Various wearable EEG devices have been proposed, such as Headset from (IMEC, 2018) (EEG Headset-IMEC), SMARTING Mobi from mBrainTrain (Mbraintrain, 2014), LiveAmp from Brain Products (Brain Products GmbH, 2015), and EEGu2 developed by Feng et al. (2016). Most of the existing solutions feature a Bluetooth-based or Wi-Fi-based wireless interface with a sampling rate of up to 1 kHz or less, and a single-chip microcomputer is generally used as the main control chip (Valentin et al., 2018; Lei et al., 2021). The wireless communication rate limits the possibility for further improvement of channel number and sampling rate. Although such configuration is sufficient for most applications of EEG, it cannot meet the requirements which arise from some practice scenarios, such as auditory brainstem response and neural spike signal acquisition.

**Figure 4:**
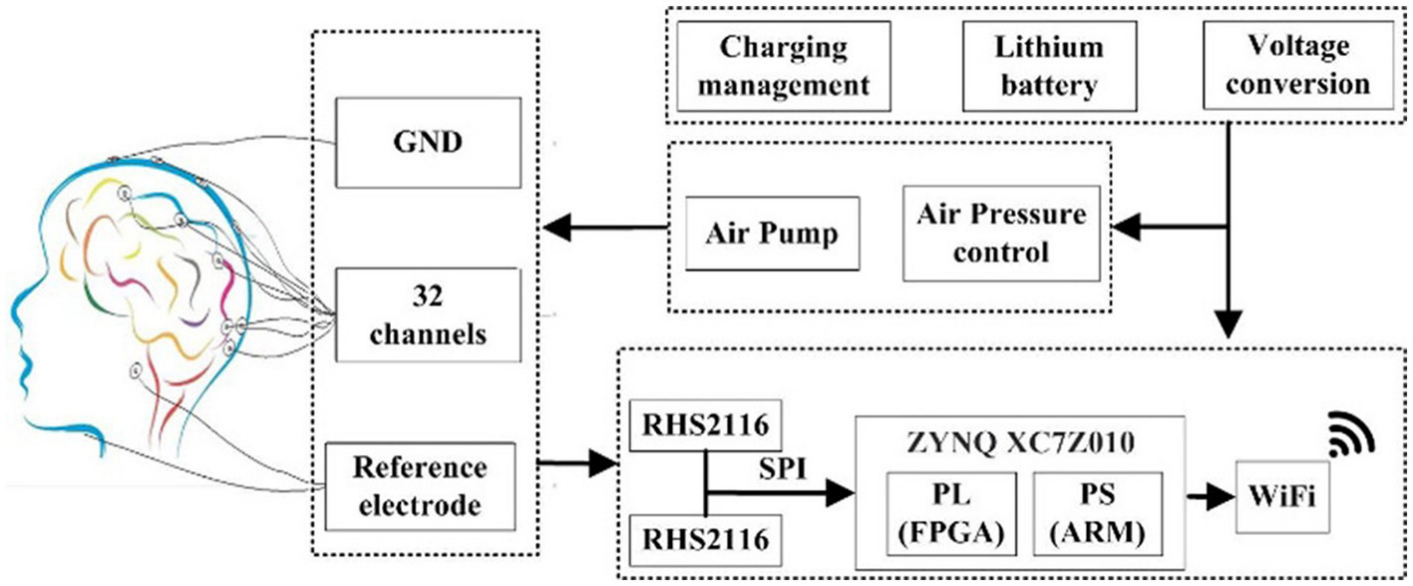
Block diagram of the designed helmet-EEG system. The biopotentials are acquired from the electrodes and amplified by the fully integrated electrophysiology microchip RHS2116, and an FPGA is used to process the information transmitted from the front-end and realize multi-channel data synchronous acquisition, and is stored in the host *via* Wi-Fi communication. The control module of air pressure can adjust the contact force between electrodes and the scalp by inflating the helmet with an air pump.

The block diagram of the proposed multi-channel helmet-EEG acquisition system is shown in Figure 4. The hardware circuit mainly includes a power supply module, air pressure control module, programmable FPGA board, and wireless communication module. The entire system employs a battery-powered circuit. The air pressure control module is designed to inflate the helmet with an air pump and to adjust the contact force between electrodes and the scalp. The fully integrated electrophysiology interface chip RHS2116 (Intan Technologies, USA) is employed for analog front-end processing and analog-to-digital conversion (ADC) of the EEG signal. The chip's internal architecture combines stimulators, amplifiers, analog and digital filters, multiplexed 16-bit ADC, and electrode impedance measurement modules on a single silicon chip. To realize high-speed data acquisition, we adopt direct memory access (DMA) mode between the ARM and FPGA. High-bandwidth wireless communication interface (Wi-Fi) is designed to transmit these data to the host. As a proof-of-concept demonstration, two chips RHS2116 are employed to construct a 32-channel parallel acquisition.

### 3.2. Implementation of high-speed parallel acquisition

**Figure 5:**
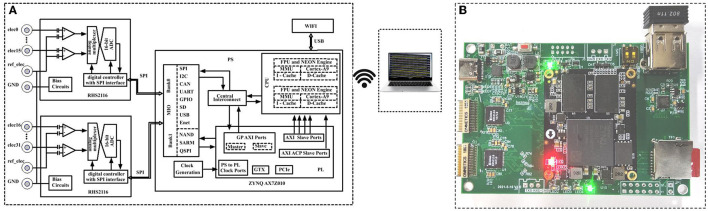
**(A)** Block diagram of the designed 32-channel EEG acquisition system and **(B)** photography of the realized circuit board.

The RHS2116 contains an array of 16 amplifier blocks, and each channel includes an AC-coupled high-gain amplifier for observing small EEG signals, as shown in Figure 5A. The on-chip registers can set the upper cutoff frequency of all amplifiers, and it is adjustable over 100 Hz to 20 kHz. The ADC may be operated at speeds up to 714 kSamples/s, which permits each channel to be sampled with 44.6 kSamples/s, fully meeting the requirement of various types of EEG acquisition, especially for the measurement and simultaneous analysis of event-related potentials. The ZYNQ XC7Z010 is employed as the kernel chip to realize stable data receiving and buffering and multi-channel synchronized receiving, and finally, the Wi-Fi module transmits the data to the upper PC for display and data storage.

The RHS2116 has a standard four-wire serial peripheral interface (SPI) with low-voltage differential signaling (LVDS) I/O pins. The LVDS signaling has the advantage of low crosstalk, constant current, and high signal integrity, which makes it far better suited for low-noise operation on a chip containing both analog and digital components. After the EEG signal passes through the ADC, the multi-channel digital signals are transferred to the FPFA chip in the ZYNQ XC7Z010 module through SPI. The ZYNQ XC7Z010 integrates a dual-core ARM Cortex A9 processing system (PS) and FPGA programmable logic (PL) on one chip, the two are interconnected through high-throughput communication, and the PS controls the PL to collect signals.

Since the FPGA and RHS2116 are directly connected through the SPI bus, whenever an SPI acquisition instruction is executed, the data from the corresponding channels of the two chips RHS2116 will be collected simultaneously, for example, channel 1 of the two chips. After the FPGA collected one channel's data, it was transmitted to the ARM through the high internal bandwidth advanced extensible interface (AXI) bus. The received data will be cached in ARM until all channels of the two chips RHS2116 are collected, and the arm processing system drives the Wi-Fi module through the USB peripheral to transmit the 32-channel data to the upper computer. It is worth noting that the RHS2116 sampling is controlled by the dynamic clock-PLL, which is different from the working clock of ARM. Therefore, a crossing clock domain asynchronous FIFO is used to transfer data between the RHS2116 module and the ARM. The AXI-DMA controller is connected to the HP port of ARM to write DDR data using the AXI4 protocol. The other end of the AXI-DMA controller reads the data from the asynchronous FIFO using the AXI4 protocol. Thus, a module is designed to encapsulate the FIFO data into the AXI4 protocol to provide data for the AXI-DMA controller.

### 3.3. Implementation of wireless communication for high-speed data transmission

The USB Wi-Fi module employed in the EEG recorder is ATK-RTL8188, which contains an ultra-low power consumption chip RTL8188EUS, a peripheral circuit, and a PCB antenna. It can be connected with other wireless devices conforming to the IEEE standard 802.11B/G/N, and the wireless transmission distance can lengthen up to 10 m. The USB Wi-Fi driver is ported into the FPGA through the Linux system and configured as a network device named WLan0. The Wlan0 works in workstation mode and automatically connects to Wi-Fi hotspots when the EEG recorder is turned on. The putty terminal connects with the EEG recorder through the serial port to test the wireless transmission rate. The experiment results show that the current design supports up to 30 kHz sampling rate for 32 channels and could support more channels as long as the required communication bandwidth does not exceed 58 Mbps (Supplementary Figure 1). Inside the RHS2116, an on-chip programmable dynamic clock is integrated, and the dynamic clock can accurately control the sampling frequency. The dynamic clock frequency follows the instructions of the upper computer to realize the dynamic adjustment of the sampling rates from 1 kHz to 30 kHz (Supplementary Figure 2). Animal experiments were also performed on rats, and spike activity was captured successfully (Supplementary Figure 3). For the developed EEG recorder, a sampling rate of 1 kHz, 64, or more channel parallel acquisition is supported. Hence, the designed system is particularly suitable for portable high-density EEG recordings, providing high spatial and temporal resolution levels.

### 3.4. Comparison between our design and several existing portable systems

The electrical circuit components are implemented on a 90 mm × 60 mm sized six-layer printed circuit board (PCB), as shown in Figure 5B. The whole circuit board is powered by a 1,000 mAh lithium battery, and the battery volume is only 60 mm × 30 mm × 3.5 mm, making it ideal for wearable devices. To achieve the connection between the 32-channel electrode and the acquisition board, an adapter board with the Omnetics A79024-1 model interface (female port) was designed. The system is also equipped with a wireless charging management circuit with functions such as constant current, constant voltage charging algorithm, current protection, and reverse discharge protection to ensure the safety of the entire system. In the case of a 30 kHz sampling rate, the system's overall operating current is approximately 800 mA, and a single charge can continuously operate for over 1.3 h. Nevertheless, reducing the sampling rate can reduce the operational burden of processing, storage, and transmission, thereby reducing the system's overall power consumption. The experiment shows that the minimum working current is approximately 500 mA.

Table 1 displays the performance of the proposed system and existing wearable EEG recorders. Due to the high-speed data processing performance of FPGA and the high bandwidth of the Wi-Fi communication module, our design is highly scalable in terms of the number of acquisition channels, supporting up to 32 or more channels of EEG data collection, and the maximum sampling rate is higher than others. Our system's EEG signal transmission rate reaches 15 Mbps, which is several times higher than others. Moreover, our helmet-EEG recorder uses dry electrodes with freely adjustable contact pressure, which can obtain better raw signals while reducing the preparation time. It is worth noting that, in the present study, we investigated the performance of the multi-channel acquisition system *via* scalp EEG, while high-density implanted electrodes can also be connected to the circuit board to realize EEG acquisition. Therefore, our proposed system's multi-channel, high sampling rate, and high data transmission rate may guarantee success in acquiring various types of EEG signals.

**Table 1:** Comparison with previous works.

	**Existing Wearable EEG Recorders**							**This study**
**References**	**(Yu et al.**, **2016****)**	**IMEC (EEG Headset-IMEC**, **2018****)**	**Smarting (Mbraintrain**, **2014****)**	**LivaAmp (Brain Products GmbH**, **2015****)**	**EEGu2 (Feng et al.**, **2016****)**	**CochlEEG (Valentin et al.**, **2018****)**	**(Lei et al.**, **2021****)**	
Channel	32	8	24	8/16/32/64	16	8	8	32
Max. sampling rate	500 Hz	1 kHz	500 Hz	1 kHz	1 kHz	4 kHz	16 kHz	30 kHz
ADC bits	24	24	24	24	24	24	24	16
Communication mode	Bluetooth	Bluetooth 2.1 EDR	Bluetooth 2.1 EDR	2.4 GHz ISM	USB (Wi-Fi)	Mini-USB	BLE 5	Wi-Fi
EEG date transmission rate	0.4 Mbps	0.2 Mbps	0.3 Mbps	1.5 Mbps	0.4 Mbps	0.8 Mbps	3.1 Mbps	15.4 Mbps
Electrode	Dry electrode	Dry electrode	Ag/AgCl	Gel-based electrode	Wet electrode	Wet electrode	Hydrogels electrode	Dry electrode
Adjustable contact pressure	Yes	Yes	No	No	No	No	No	Yes

## 4. Experimental results

### 4.1. Basic signal measurement and evaluation

**Figure 6:**
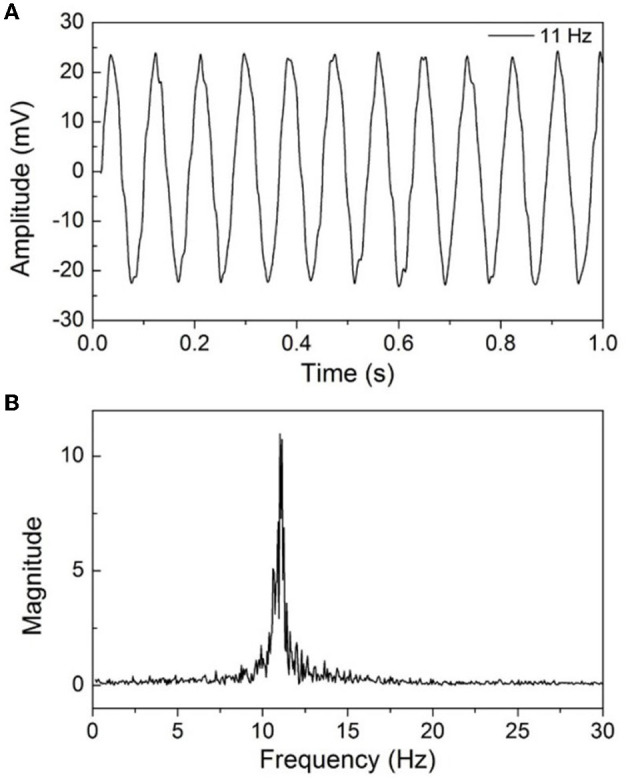
**(A)** Basic electrical test results of the proposed EEG recorder with sinusoidal signal operating at 11 Hz, **(B)** the frequency spectrum of the collected signal.

The EEG signal amplitude is generally in the range of 10–100 μV, and we first test the feasibility of the developed EEG recorder by a sinusoidal signal. The input signal is provided by a programmable function generator and then attenuated through a voltage-dividing resistor to obtain a microvolt signal. The amplitude of the input signal is approximately 2.5 mV, and the signal frequency is 11 Hz; it was input to the RHS2116 after 10 times attenuation. Figure 6A shows the waveforms of the collected signal after a low pass filter with a 50 Hz cutoff frequency was used, and Figure 6B shows the frequency domain spectral. It can be seen that the time domain signals show high SNR and stability, and the frequency is consistent with the expectation. The slight top distortion of time domain signals may be caused by noise and external interference. As the input signal was attenuated 10 times and then amplified by the RHS2116 with a gain of approximately 190, the amplitude of the collected signal is theoretically 19 times higher than the input signal.

**Figure 7:**
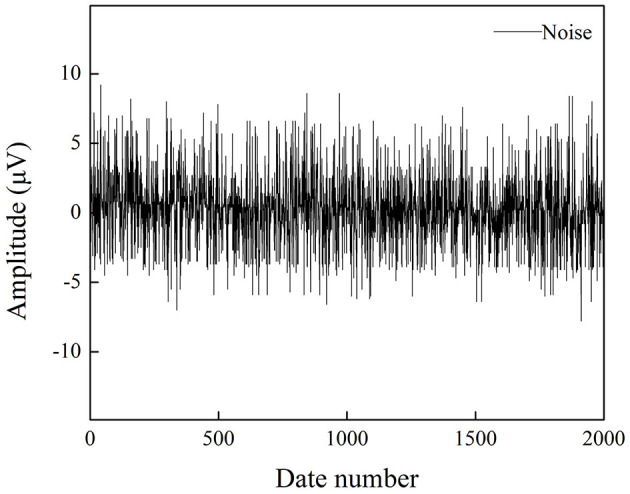
Input noise test result of the proposed EEG recorder.

Noise is an essential criterion for evaluating EEG acquisition equipment, including the internal noise of the acquisition chip and the overall circuit. The noise level directly affects the signal quality, especially for weak signals. To validate the performance of the developed EEG recorder, the noise signal was collected when input was shorted. In the measurement, the sampling rate was set to 1 kHz. Figure 7 shows the noise waveform within 2 s, and the amplitude is within the range of 8 μV. The root mean square (RMS) of the input noise can be calculated by the following formula:


$$\begin{array}{l} u=\sqrt{\frac{\sum_{i=1}^{n}x_{i}^{2}}{n}} \end{array}$$

The value is calculated to be 2.62 μV, and the testing results closely achieve the low-noise characteristics of 2.4 μVrms as specified in the datasheet of the RHS2116 chip, proving the ability of the proposed EEG recorder to acquire weak biopotentials.

### 4.2. Measurement of the skin-electrode impedance

**Figure 8:**
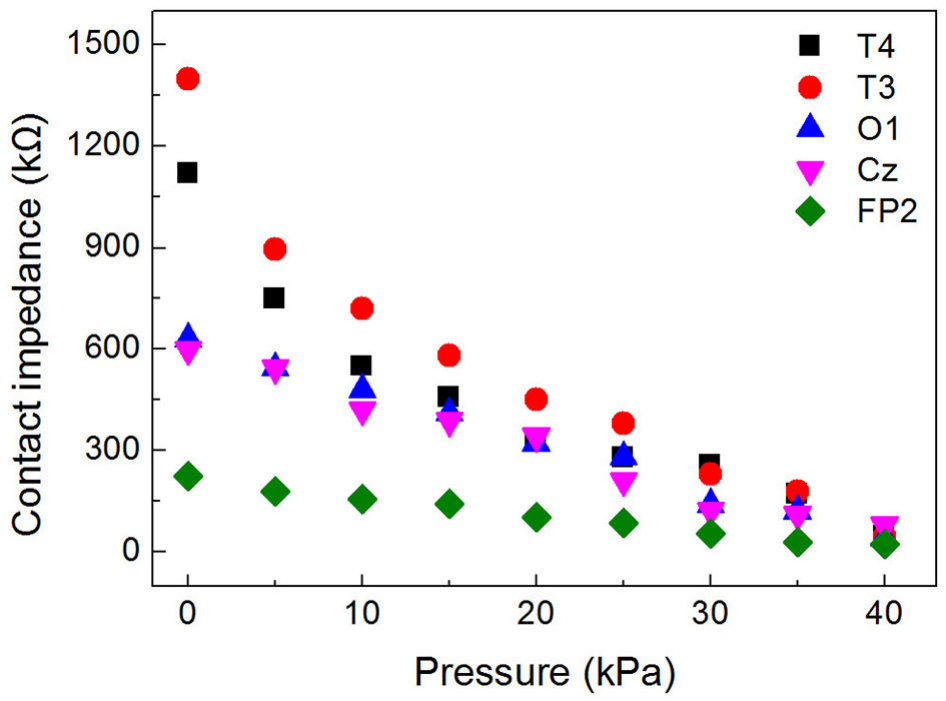
Dry electrode-scalp impedance obtained at different positions under different pressure.

The inflatable helmet-EEG recorder proposed in this study could increase the pressure between the electrode and the scalp by increasing the airbag pressure, thereby reducing the contact impedance and improving the quality of the EEG signal. For the experiment, a 24-year-old male subject wore the EEG helmet and inflated the helmet with different pressures. The FP2, Cz, O1, T3, and T4 channels were selected as the working electrode, and the adjacent electrode was selected as a reference electrode. The skin-electrode impedance was measured at 1 kHz by an impedance analyzer (TH2838A, Tonghui). The results of the experiment are displayed in Figure 8.

As can be seen, when the EEG helmet was not inflated, the contact impedance between the dry electrode and scalp was about several hundreds of kΩ, and the value of the Cz channel was 598 kΩ, much lower than that of T3 and T4, whose values are 1,400 and 1,120 kΩ, respectively. This is because the helmet's gravity produces pressure on the overhead area and reduces the contact impedance in the top-down direction. The contact impedance at FP2 was 223.9 kΩ, much lower than other positions due to the forehead region having less hair. In addition, the contact impedance showed a decreasing trend with increasing pressure. Figure 8 shows that the value was 23.6, 80.5, 40.4, 45.6, and 47.8 kΩ for FP2, Cz, O1, T3, and T4 channels, respectively, when the pressure of the EEG helmet reached 40 kPa, and the averaged value was 47.6 kΩ, only 1/16 of that before inflation, suggesting that the proposed EEG helmet could achieve good contact between the dry electrode and the scalp.

### 4.3. EEG measurement experiment

**Figure 9:**
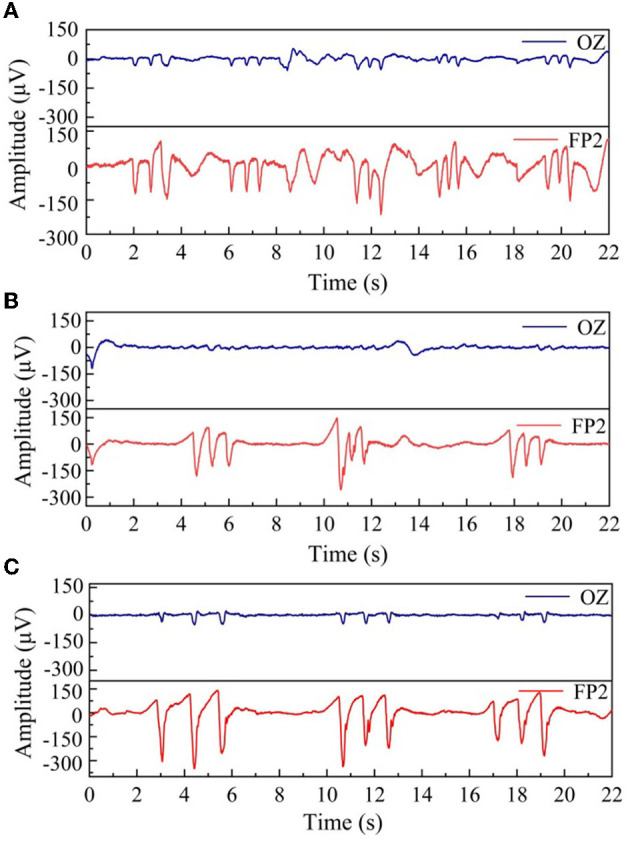
Comparison of three groups of consecutive blinking signals with different air pressure values, **(A)** 5 kPa, **(B)** 20 kPa, and **(C)** 40 kPa.

In this section, we inflate the helmet to 5 kPa, 20 kPa, and 40 kPa and collect the corresponding EEG signals from the forehead FP2 and the occipital region Oz. In the measurement, the two reference electrodes were placed under the earlobe by clamp, and the sampling rate was also set to 1 kHz. The EEG signals due to natural blinking are shown in Figure 9. Figures 9A–C is the blink signal collected under the inflation pressure of 5 kPa, 20 kPa, and 40 kPa, respectively. It can be seen from the figure that when the air pressure reaches 5 kPa, the signal fluctuates wildly and is accompanied by interference. When the air pressure increases to 40 kPa, the blinking signal collected by Oz and FP2 is more apparent, and the blinking signal is clearer with reduced noise. We selected a stable segment of the FP2 channel as the noise and calculated the SNR; the results show that the SNR was 44, 116, and 159, respectively, so the pressurization can effectively improve the SNR.

**Figure 10:**
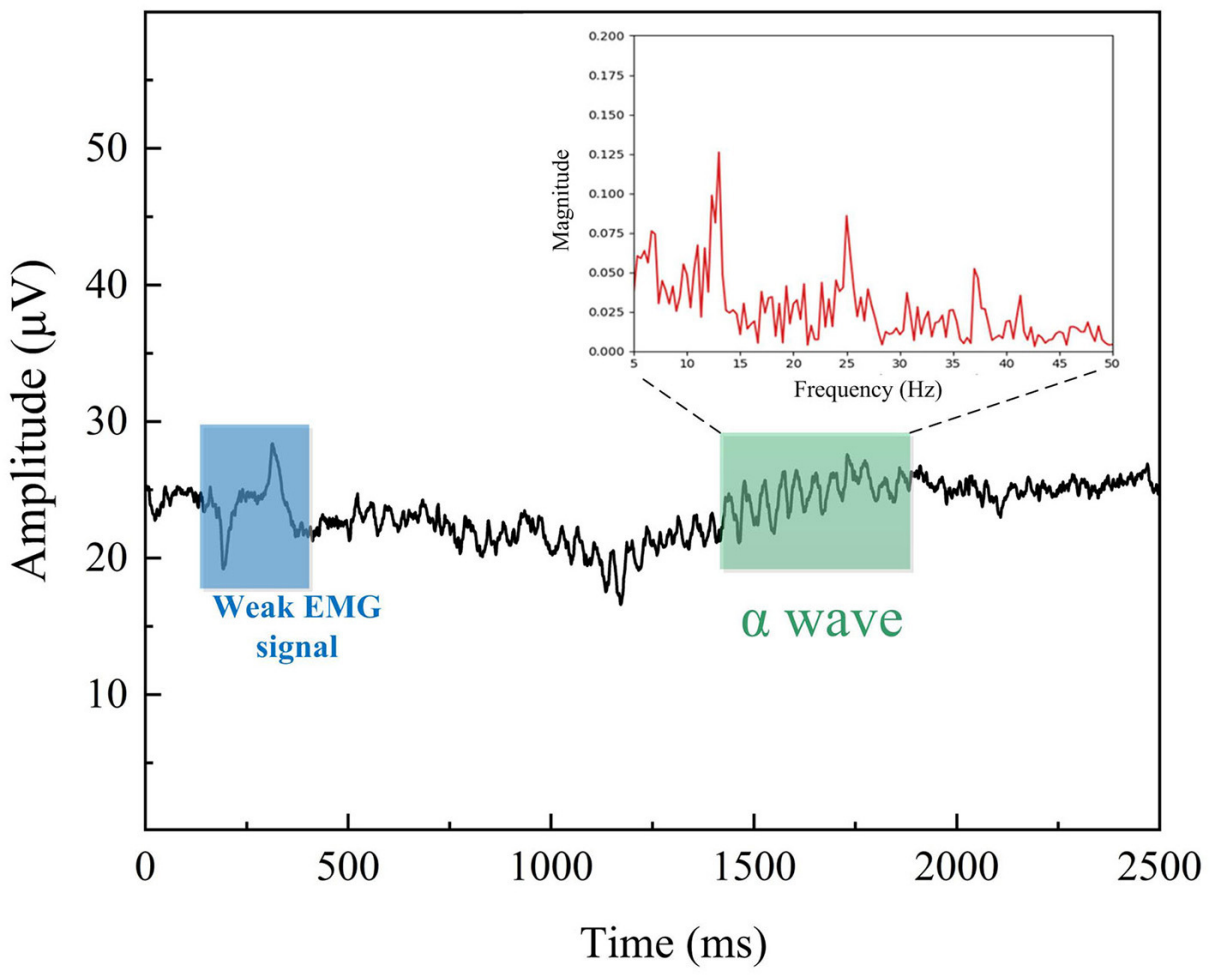
EEG signal of Oz channel.

Alpha (α) rhythm refers to the 8–13 Hz rhythmic α-waves recorded in the occipital region when subjects are awake with their eyes closed, and we performed a typical α rhythm test to prove the EEG helmet feasibility. As the electrode sites on the posterior regions such as Oz positions could obtain a better α-wave signal, the Oz channel EEG signal was collected and analyzed. The reference point and GND point are connected to the lower part of the earlobe of both ears using Ag/AgCl electrodes. In a quiet indoor environment, the subject leaned back on the seat, adopted a sitting posture, relaxed all body muscles, and performed a single blink test. The obtained typical raw EEG signal without a bandpass filter is shown in Figure 10. As displayed in the figure, the back pillow generates a slight EMG signal when the subject blinks, and the time domain waveform of the EMG signal is marked in the blue box, indicating that the subject had blinked his eye at this time. Additionally, we observed that the α-wave began to appear after 1.5 s, and the typical spindle-like waveform of the α-wave can be clearly seen, as the region marked with the green box. The corresponding frequency spectrum shows that the magnitude in the 8–13 Hz frequency band is relatively large and consistent with expectations. The feasibility test indicates that the helmet-EEG recorder can capture the bioelectric signals during everyday activity without noticeable interference.

### 4.4. The SSVEP experiment

In this study, the classical paradigm was employed to examine the feasibility of the developed inflatable helmet-EEG recorder in BCI applications. SSVEP is an evoked potential generated by presenting flash or pattern stimulation; it is a periodic electrical response of the brain to sensory stimulation. The SSVEP responses usually originate from the occipital cortex, which is the brain region involved in signal reception and interpretation of visual signals, and it can be obtained from the electrode placed at the back of the brain.

**Figure 11:**
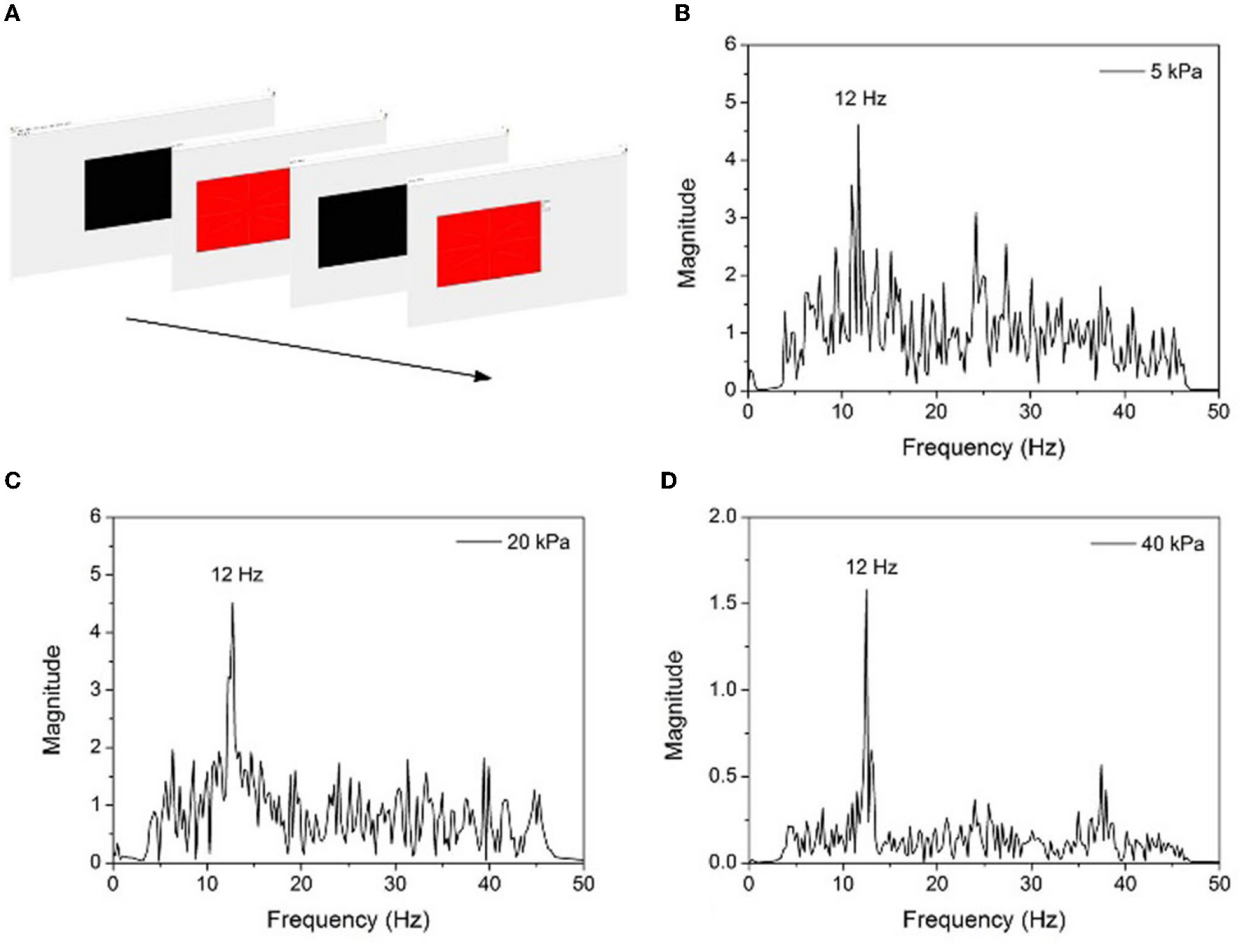
**(A)** SSVEP experiment design diagram, **(B)** SSVEP power spectrum under 12 Hz stimulation and 5 kPa inflation pressure, **(C)** 20 kPa, and **(D)** 40 kPa.

When stimulating the retina in the range of 3.5–75 Hz, the brain responds in the same way as visual stimuli frequency (or multiple) and generates electrical activity. The target of stimulation can be determined by detecting the SSVEP signal. In the experiment, red square image with a flicker frequency of 12 Hz was presented on a 17-inch computer screen with a 60 Hz refresh rate. The experiment was conducted in a room without light, and the subjects looked at the display 60 cm away, randomly stared at the stimulus image for 60 s, and repeated five times to ensure the reproducibility of the result. After 50 Hz notch filter and bandpass filter processing, the offline data of the Oz channel EEG signal (Supplementary Figure 4) were collected and transformed into a power spectrum employing fast Fourier transform (FFT) functions. Three groups of experiments were conducted under different inflation pressure. The results are shown in Figure 11. It can be seen that the SSVEP power spectrum has a prominent peak at the stimulation frequency, and it appears more evident with the increase of inflation pressure. The ratio of noise power at 12 Hz and 30 Hz increased from 3 (5 kPa) to 8 (40 kPa), demonstrating the effectiveness of improving EEG signal quality by air pressure regulation of the helmet.

**Figure 12:**
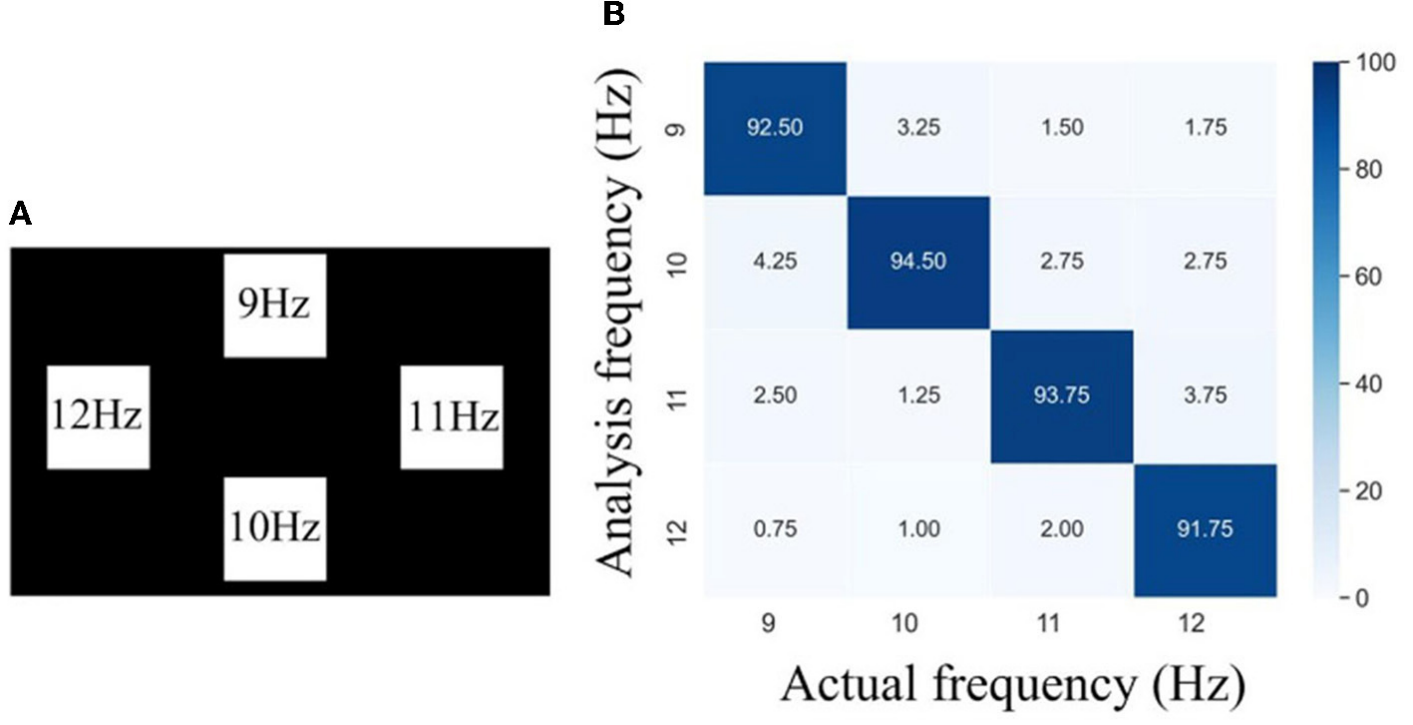
**(A)** Stimuli design, and **(B)** confusion matrix of visual stimulus frequency and recognition frequency.

To further investigate the effectiveness of the helmet–EEG system for SSVEP-based BCI, targets with different flicker frequencies were presented on the screen and employed to stimulate the brain. Figure 12A shows the distribution and frequency values of the stimulus targets. In this experiment, the subject wore the EEG helmet with an inflation pressure of 40 kPa, paid attention, and gazed alternately at a set of 4-targets whose flicker frequencies were set at 9 Hz, 10 Hz, 11 Hz, and 12 Hz, respectively. Each trial included 5 s stimulus and 1 s cue, which was then switched to another stimulus frequency. Each subject carried out 10 trials of recording. Between the recordings of each trial, the subject took a break for 1 min. The entire recording was used for the calculation of the frequency response. Six channel (Pz, P3, P4, Oz, O1, and O2) EEG signals were collected over the occipital and parietal areas. The recorded signals were filtered through 1-Hz high-pass and 50-Hz low-pass filters first and then wirelessly transferred to a laptop computer *via* Wi-Fi. The captured EEG data were imported into MATLAB for offline analysis. We used a convolution neural network (CNN) proposed by Liu X. et al. (2021) for feature extraction and classification of the evoked SSVEPs. We tested our system for a total of 50 trials in five male subjects to obtain the overall accuracy, and all subjects were healthy and had no record of neurological and psychiatric disorders. Each subject was given a summary of the experiment and signed a consent form before their participation started. The confusion matrix of stimulus frequency and recognition frequency is presented in Figure 12B. It can be found that there is a significantly high correlation on the diagonal, and it is in line with the theoretical analysis of SSVEP. The average accuracy of SSVEP identification was 93.12%, and the minimal and maximal accuracy were 91.75 and 94.5%, respectively, proving the correctness of the results of the inflatable helmet-EEG system and showing the feasibility of the SSVEP-based BCI system.

## 5. Conclusion

We developed an inflatable helmet-EEG system, which can collect EEG signals in a convenient and comfortable manner. For the first time, a high-speed EEG recorder was realized using a fully integrated electrophysiology microchip RHS2116, a high-bandwidth Wi-Fi communication module, and an FPGA chip. The inflatable helmet-EEG recorder has 32 channel standard electrode distribution, provides a maximum 30 kHz sampling rate and 16-bit accuracy and a 58 Mbps wireless transmission rate that meets the requirements of a high sampling rate, and is compact in size and comfortable for wearable scalp EEG and intracranial EEG collection. The contact impedance between the electrode and the scalp can be adjusted by regulating the inflation pressure of the airbag. Through a series of experiments, the applicability of the developed system has been successfully demonstrated for EEG recording and SSVEP detection, and it is expected to promote the application of EEG signals on the SSVEP-based BCI system.

## Data availability statement

The original contributions presented in the study are included in the article/Supplementary material, further inquiries can be directed to the corresponding authors.

## Ethics statement

The studies involving human participants were reviewed and approved by Affiliated Hospital of Hebei University. The patients/participants provided their written informed consent to participate in this study.

## Author contributions

RL, YZ, CL, and SF carried out the design of the experiment. RL and YZ implemented the experiment. RL and GF accomplished the data processing. RL and CL wrote the manuscript. CL, JL, and XL checked and modified the manuscript. All authors read and approved the final manuscript.
