# Supplementary material for: Design and implementation of high sampling rate and multichannel wireless recorder for EEG monitoring and SSVEP response detection
Source: Front Neurosci. 2023 Jun 29;17:1193950. doi: 10.3389/fnins.2023.1193950 (PMC10339741; doi:10.3389/fnins.2023.1193950)
Supplement: Supplementary file 1 [file Data_Sheet_1.docx]

**SUPPLEMENTARY MATERIAL**

**Design and Implementation of Inflatable Helmet-EEG Wireless Recorder with Application to SSVEP Detection**

Ruikai Li^†^, Yixing Zhang^†^, Guangwei Fan, Ziteng Li, Jun Li, Shiyong Fan, Cunguang Lou*, and Xiuling Liu*

The following steps were conducted to evaluate the wireless communication bandwidth of the EEG recorder, and the results were shown in the supplementary Fig. 1. As can be seen from the figure, the average wireless transmission speed is 57.9 Mbits/s.

Test steps:

1. Run iperf: iperf3-s on the computer side;

2. Use the putty terminal to connect the collection device through the serial port, and log in to the system as root: root;

3. Run iperf at the putty terminal: iperf3- c 192.168.0.6

4. Record the iperf test results.


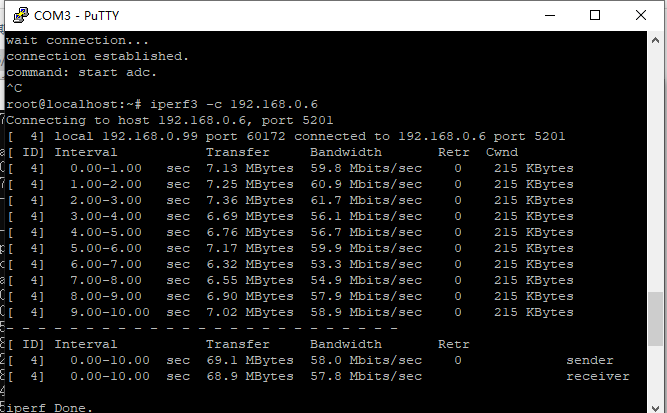


Fig. 1 Testing transport performance of the wireless WiFi module of the EEG recorder.

The sampling rate of the EEG recorder was measured by collecting standard sinusoidal signals. Configure the signal source to generate a sine wave signal with a frequency of 20 Hz and amplitude of 2 mV. Then start the acquisition and transmission equipment and make it work at a sampling rate of 30 k/s, wait until the network of acquisition equipment is ready, run the EEG data waveform viewing software at the computer end, and click the "Start" button to start the acquisition. Fig. 2 shows one cycle of the signal collected by the EEG recorder, its period is 1/20 s, and the number of data points is 1500, so the sampling rate is 1500/0.05s=30 kHz.


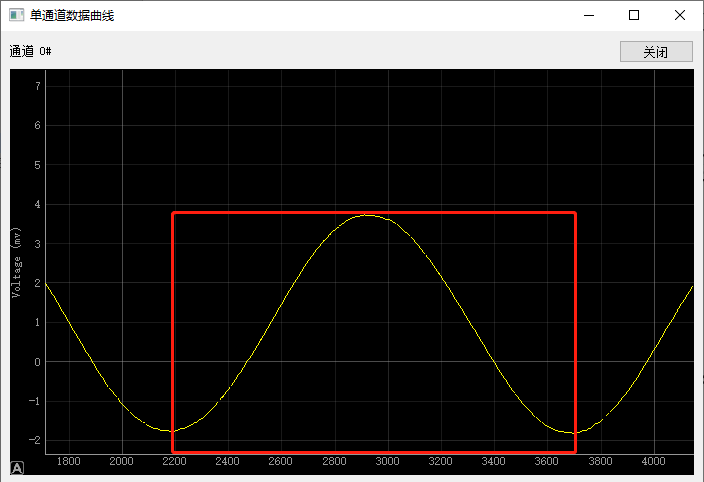


Fig. 2 The captured 20 Hz-sinusoidal signals by the developed EEG recorder.

To verify the feasibility of the system for spikes signal collection, experiment was performed on a rat. The experimental and surgical procedures were approved by the Institutional Animal Care and Use Committee at the Chinese Academy of Military Medical Science. Fig. 3 shows the photography of experiments and the collected signals, as we can see, the figure illustrates that the ECoG wave contains spikes signal with amplitudes far higher than the background noise. Additionally, the spike signals have a high frequency and appear for a brief duration with a large peak and several smaller peaks. The results were in good condition, with stable signals and no noise, meeting the signal collection requirements for high sampling rate.


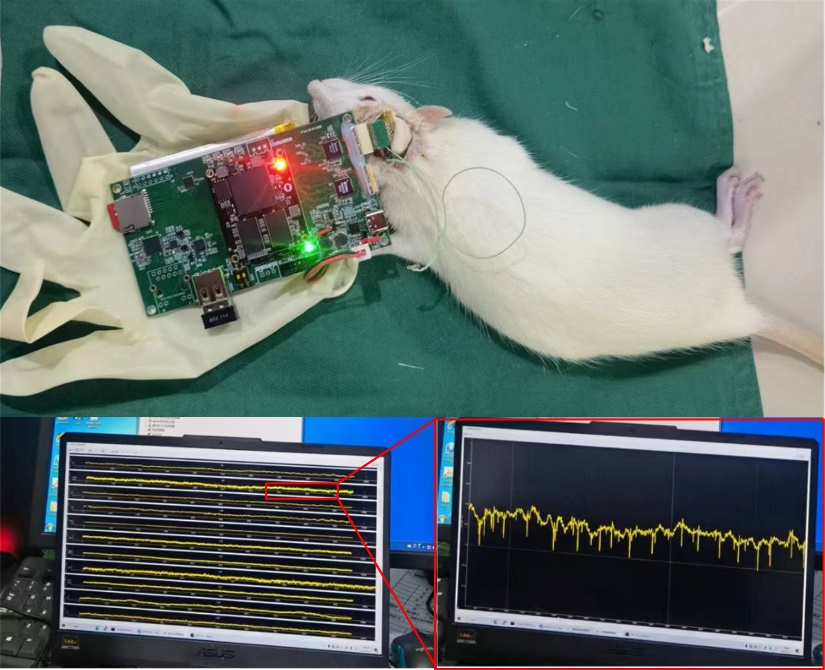


Fig. 3 The acquisition experiment of rat ECoGs and the captured neural spikes signal.

The red squares image with a flicker frequency of 12 Hz was presented on a 17-inch computer screen with a 60 Hz refresh rate. The experiment was conducted in a room without light, and the subjects looked at the display 60 cm away and randomly stared at the stimulus image for 60 seconds. After 50 Hz notch filter and bandpass filter processing, the time domain EEG signal of OZ channel was collected and displayed in Fig. 4. As can be seen from the figure, with the increase in inflation pressure, the SSVEP signal is more evident with improved signal-to-noise ratio.


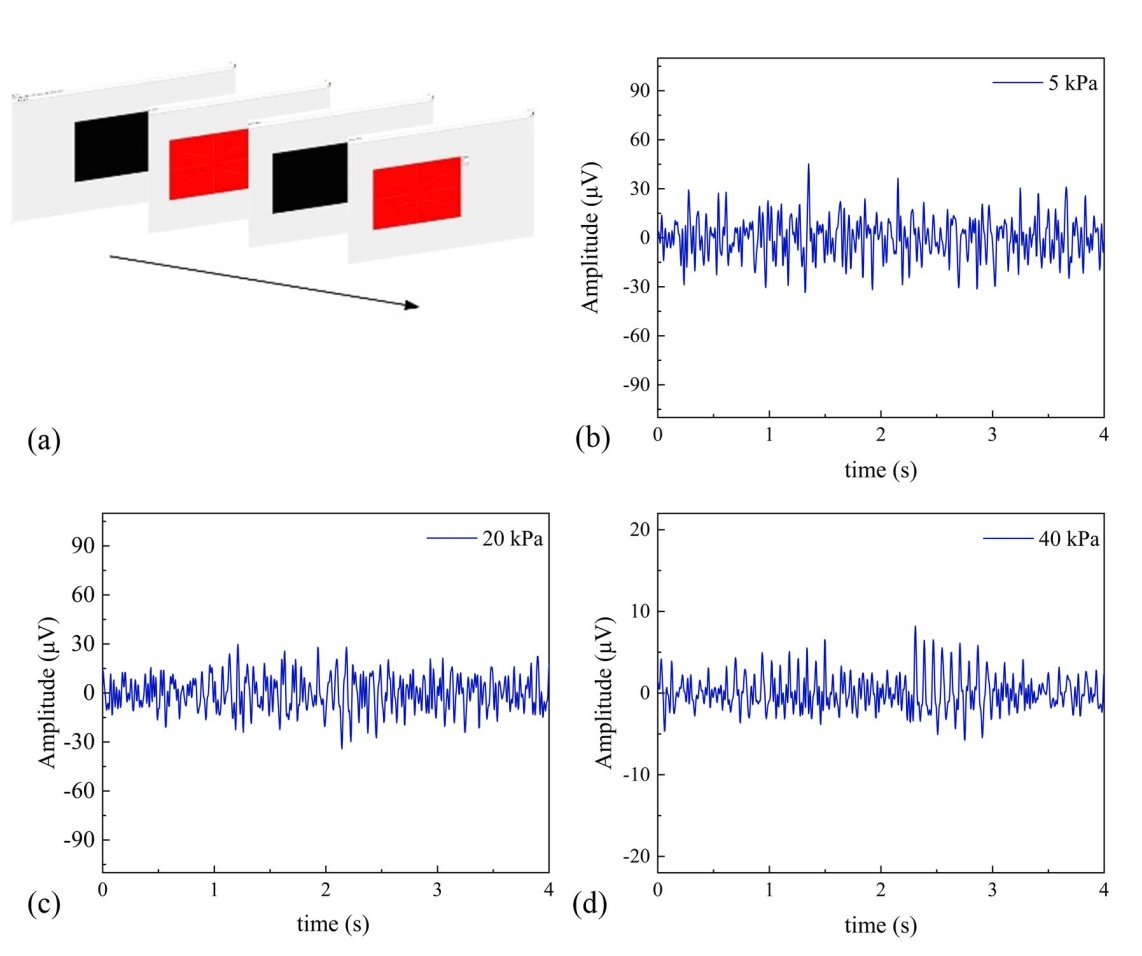


Fig. 4 The captured SSVEP signal by the EEG helmet under different inflation pressure.
